# A toolbox and sample object perception data for equalization of natural images

**DOI:** 10.1016/j.dib.2015.10.030

**Published:** 2015-11-06

**Authors:** Wilma A. Bainbridge, Aude Oliva

**Affiliations:** a The Department of Brain and Cognitive Sciences, Massachusetts Institute of Technology, 77 Massachusetts Avenue, Cambridge, MA 02139, United States; b Computer Science and Artificial Intelligence Laboratory, Massachusetts Institute of Technology, 77 Massachusetts Avenue, Cambridge, MA 02139, United States

**Keywords:** Natural image statistics, Spatial frequency, Object interaction envelope, Object perception

## Abstract

For psychologists and neuroscientists, careful selection of their stimuli is essential, so that low-level visual features such as color or spatial frequency do not serve as confounds between conditions of interest. Here, we detail the *Natural Image Statistical Toolbox*, which allows scientists to measure, visualize, and control stimulus sets along a set of low-level visual properties. Additionally, we provide a set of object images varying along several perceptual object properties, including physical size and interaction envelope size (i.e., the space around an object transversed during an interaction), serving as a test-bed for the Natural Image Statistical Toolbox. This stimulus set is also a highly characterized set useful to psychology and neuroscience studies on object perception.

**Specifications table**

**Table:** 

Subject area	*Psychology*
More specific subject area	*Vision and Perception*
Type of data	*Code, visual stimuli, and a spreadsheet describing characteristics of the visual stimuli*
How data was acquired	*Code, image database*
Data format	*MATLAB code, JPEG images, .XLS/.CSV spreadsheet*
Experimental factors	*Stimuli were obtained from an in-lab object image database*
Experimental features	*Stimuli characteristics were obtained from subject ratings on various object properties in an Amazon Mechanical Turk study*
Data source location	*Cambridge, MA*
Data accessibility	*Data will be linked to from this article.*
*Stimuli with characteristics spreadsheet:*
*http://wilmabainbridge.com/interactionenvelope.html*
*Natural Image Statistical Toolbox:*
*http://wilmabainbridge.com/naturalimagestatstoolbox.html*

**Value of the data**

• The *Natural Image Statistical Toolbox* will allow psychologists and neuroscientists to test their stimuli for low-level visual statistics between their conditions.
• This toolbox will be constantly evolving and updated with other scripts as more is understood about visual processing, so that scientists can fine-tune the best natural stimuli for their experiments.
• The *Object Interaction Envelope Stimuli* set is the first stimulus set measuring objects along the property of “interaction envelope”. With this highly characterized set (for both low-level visual and higher-level object properties), cognitive scientists can conduct their own experiments on object interaction envelope.
• This stimulus set also serves as a test-bed for the *Natural Image Statistical Toolbox*, for experimenters to try out the scripts.

## 1 Data

The data consist of two main components: 1) The *Natural Image Statistical Toolbox*, and the 2) *Object Interaction Envelope Stimuli* set.

## 2 Experimental design, materials and methods

### 2.1 Natural image statistical toolbox

It is often difficult to only manipulate a specific parameter of interest when designing psychophysics or neuroimaging experiments, as visual properties are often intercorrelated with other perceptual properties. For example, in selecting stimuli of large and small real-world physical size [1], these stimuli will also often differ in terms of visual properties such as spatial frequency information (i.e., larger objects have more detail, and thus higher spatial frequencies) or color information (i.e., large objects may be more monochromatic).

**Fig. 1:**
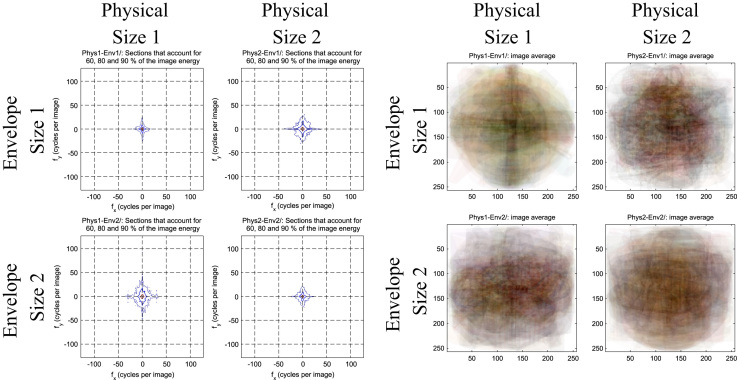
An example of how one might use the *Natural Image Statistical Toolbox* to compare stimulus conditions (in this case, the conditions from the *Object Interaction Envelope Stimuli set*). (Left) Visualizations of spectral energy levels of the different conditions orthogonalizing object physical size and envelope size [1]. As one can see, there are subtle differences in the spatial frequency information across the conditions. The *Natural Image Statistical Toolbox* also outputs statistical information and performs *t*-tests between relevant conditions. In this case, there were no significant differences in spatial frequency for comparisons of interest (i.e., between orthogonalized conditions, and between intercorrelated conditions). (Right) The image average of all of the stimuli for each condition. With this, one can assess condition differences in shape, spatial frequency information, color, orientation, amount of white space, etc.

To address this issue, a set of MATLAB scripts (The *Natural Image Statistical Toolbox*) was developed based on previous work looking at natural image statistics [2] for the purpose of measuring and controlling a set of simple low-level visual confounds in stimuli for psychophysics and neuroimaging experiments (see Fig. 1 for some examples). Currently the toolbox consists of four main components, but will be dynamically updated as other stimulus-controlling scripts are developed:

1) Spatial frequency scripts – these scripts measure, visualize, and statistically compare spatial frequency (spectral energy) information in image sets. These are particularly useful for controlling stimuli for activation in the early visual cortex, and have been successfully used to equalize stimuli and diminish V1 activity in a scene perception experiment [3].
2) Color histogram scripts – these scripts measure, visualize, and statistically compare color distribution information in image sets. This works in both RGB color space as well as Lab color space, and can look at both brightness and contrast. These are useful for controlling for color-related activation in the early visual cortex and IT.
3) Isolated image space scripts – scripts to measure, visualize, and statistically compare amounts of non-white space in images of objects against isolated white backgrounds. Essentially, this quantifies the retinal size taken up by each stimulus.
4) Permutation test script – a script that does a non-parametric permutation statistical test. While this is used here for comparing non-unimodal color histogram distributions, this could be easily adapted for other non-parametric testing.

The toolbox is maintained and updated at the author’s site:

http://wilmabainbridge.com/naturalimagestatstoolbox.html

### 2.2 Object interaction envelope stimuli

Included with the *Natural Image Statistical Toolbox* is a set of example stimuli: the *Object Interaction Envelope Stimuli* set. This stimulus set was developed in order to allow cognitive scientists to explore the novel perceptual object property of interaction envelope [1], using a highly characterized stimulus set. Interaction envelope refers to the space through which one transverses to interact with an object, and is operationalized here as the number of hands most often used to interact with an object, resulting in two very different, non-overlapping volumes of interactive space. We anticipate this stimulus set will be useful both to researchers in object perception who wish to quantify interaction envelope, as well as experimenters wanting a proven set of test stimuli for the *Natural Image Statistical Toolbox*.

### 2.2.1 Stimulus images

**Fig. 2:**
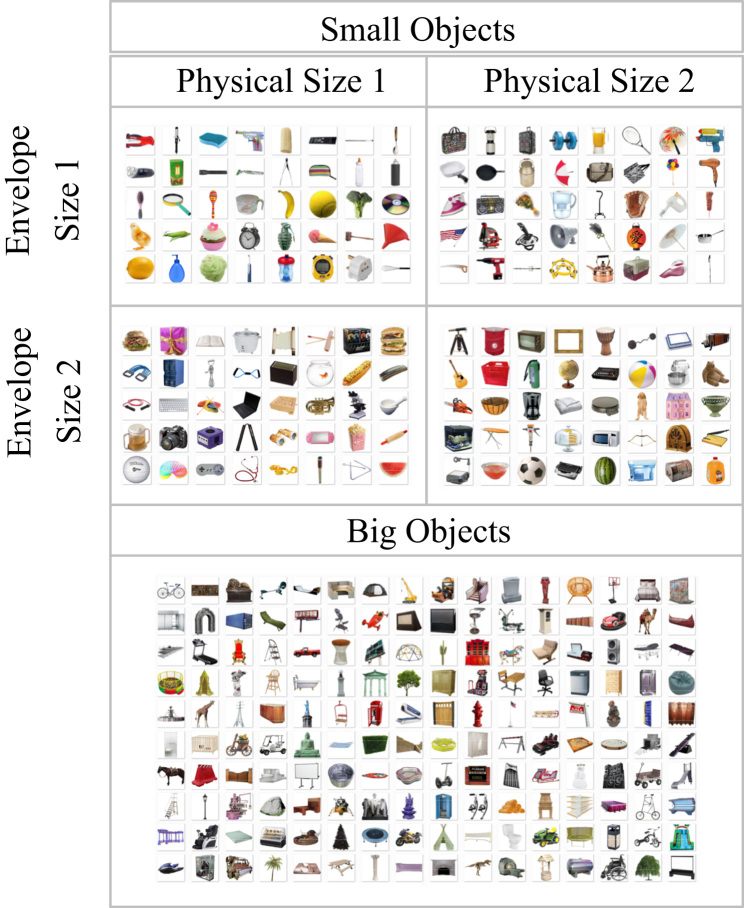
The 360 object images included in the *Object Interaction Envelope Stimuli* set. Object properties for all small-scale object images are also included.

The stimulus set includes 320 images of isolated objects against a white background, where 160 of the images are large-scale objects of both big physical size and interaction envelope, and 160 are images of small-scale objects (see Fig. 2). Within the small-scale object set, there are four smaller conditions with 40 images each that orthogonalize real-world physical size and object interaction envelope: 1) objects of both small physical size and envelope size, 2) objects of both large physical size and envelope size, 3) objects of small physical size and large envelope size, and 4) objects of large physical size and small envelope size. Images are all JPEG images of 350×350 pixels in size. Using the *Natural Image Statistical Toolbox*, these images were selected so that the four conditions did not differ in average RGB color, amount of white space, luminance, or spatial frequency information.

### 2.2.2 Stimulus characteristics

The small-scale object stimuli were characterized on several perceptual object properties described below, identified in modulating activity within the scene-selective parahippocampal place area (PPA; [4]). These characteristics were collected from a series of Amazon Mechanical Turk (AMT) experiments asking participants to rate the images on various characteristics. All stimulus characteristic information is available in the XLS and CSV files included with the stimuli.

#### 2.2.2.1 Participants

     Ultimately 431 individuals participated from AMT and consented to participation based on the guidelines of the MIT Committee on the Use of Humans as Experimental Subjects. Participants were screened so they had at least a 95% AMT approval rating, and were paid at a rate of approximately $5/h. Each object property was rated by fifteen participants per image, although participants were allowed to respond on multiple objects.

#### 2.2.2.2 Physical size

      The real-world size of an object has been found to be linked with activity differences in the parahippocampal cortex and transverse occipital sulcus [5]. For the current stimulus set, object physical size was characterized in two different ways. First, ground-truth estimates were determined by shipping sizes for each object from the online marketplace Amazon.com (and other similar marketplaces). Included in the data are the shipping length (in), width (in), height (in), volume (in3), longest dimension (in), three-dimensional diagonal (in), and weight (lbs).

Second, subjective estimates for size were taken on AMT, where participants were asked to rate each object’s size and weight on a Likert scale of 1–7, and were given examples where 1 would be close to the size and weight of a pushpin, while 7 would be close to the size and weight of a large monument.

#### 2.2.2.3 Handedness

     To confirm the number of hands, and thus the interaction envelope, for each stimulus, participants were asked how many hands they use when they first interact with each given object.

#### 2.2.2.4 Fixedness

     Fixedness of an object in an environment has been found to correlate with PPA activation [6]. Using the same methods as [6], participants were asked to rate on a 5-point Likert scale how easily they could pick up and move each object.

#### 2.2.2.5 Placeness

     Placeness of an object measures the degree to whether people classify a given stimulus as either a place or a thing, on a binary scale [7]. Participants were asked to determine if each given image was more like a place or thing.

#### 2.2.2.6 Spatial definition

      Spatial definition is another property shown to modulate PPA activation [8]. Participants were given the following instructions to determine spatial definition of each object: “We call an object a ‘space defining object’ when it evokes a strong sense of surrounding space and is hard to imagine in isolation. In contrast, we call an object a ‘space ambiguous’ object when it *does not* give a feeling of space around it but is easy to imagine in isolation. Is this more of a space defining object or a space ambiguous object?” Participants then gave a binary answer.

#### 2.2.2.7 Context

     Context is defined as the degree to which there is a consistent environment an object occurs in [9]; for example, a frying pan will almost always appear in a kitchen, while a pen could be in any number of environments (e.g., office, bedroom, library, classroom). Participants were asked to briefly state where they would normally find each object. An entropy score was then calculated across all the responses to indicate degree of context:

E=∑p(x)×log(p(x))

Here, x is all different answers and p(x) is the proportion of responses that are x [7], [9]. When using fifteen participants per object, this score ranges from −1.18 (where all 15 responses are different) to 0 (where all 15 responses are the same).

The responses provided have already been hand-coded from the text responses to numerical labels for ease of use (as the text responses varied in terms of spelling, synonym usage, etc). For example, 5 responses like {“the kitchen”, “livingroom”, “KITCHEN”, “living room”, “dining room”} would be coded as [1, 2, 1, 2, 3].

The Object Interaction Envelope stimuli images (Section 2.1) as well as characteristic spreadsheets (Section 2.2) can be accessed on the author׳s website:

http://wilmabainbridge.com/interactionenvelope.html
